# Dr. Robert Smith

**Published:** 1910-06-04

**Authors:** 


We have to record the death of Dr. Robert Smith, M.D., late medical superintendent of Durham County Asylum, in his seventy-eighth year. Dr. Smith took his M.B. at King's College, Aberdeen University, in 1854, and his M.D. at the same University in 1858. Among his early appointments we may notice that of assistant medical officer to the Southern Counties Asylum, Dumfries, and that of medical superintendent of the Bath Lane Asylum, Newcastle-on-Tyne.

